# Perceiving emotions in visual stimuli: social verbal context facilitates emotion detection of words but not of faces

**DOI:** 10.1007/s00221-020-05975-9

**Published:** 2020-11-18

**Authors:** Stephanie S. A. H. Blom, Henk Aarts, Gün R. Semin

**Affiliations:** 1 Department of Psychology, Utrecht University, Utrecht, The Netherlands; 2 Department of Psychology, ISPA, William James Center for Research, Instituto Universitário, Lisbon, Portugal

**Keywords:** Emotion detection, Emotion processing, Auditory context, Words and faces, EMG

## Abstract

Building on the notion that processing of emotional stimuli is sensitive to context, in two experimental tasks we explored whether the detection of emotion in emotional words (task 1) and facial expressions (task 2) is facilitated by social verbal context. Three different levels of contextual supporting information were compared, namely (1) no information, (2) the verbal expression of an emotionally matched word pronounced with a neutral intonation, and (3) the verbal expression of an emotionally matched word pronounced with emotionally matched intonation. We found that increasing levels of supporting contextual information enhanced emotion detection for words, but not for facial expressions. We also measured activity of the corrugator and zygomaticus muscle to assess facial simulation, as processing of emotional stimuli can be facilitated by facial simulation. While facial simulation emerged for facial expressions, the level of contextual supporting information did not qualify this effect. All in all, our findings suggest that adding emotional-relevant voice elements positively influence emotion detection.

## Introduction

A considerable part of peoples’ everyday lives consists of accurately grasping emotion-related information. In social interactions, emotion-related information can come in different forms and shapes. People might use language to convey affective information or to communicate their emotional states, such that emotion processing is directed at verbally expressed or written words. In addition, social interaction involves emotional facial expressions that can reveal the emotional states of interaction partners.

Understanding and accurately detecting the emotionality of communicated information that is targeted in social interaction does rarely happen in isolation. Often, emotional processing is accompanied by context (e.g. Arnold and Winkielman 2019; Aviezer et al. 2017; Feldman Barrett et al. 2011; Mermillod et al. 2018) that can support the identification of emotionality of target information. For instance, consider a common read-aloud setting in which a child is taught to read a book. In such a situation, the child not only sees the words, but also might hear the very same words and intonation with which these words are being articulated by a caregiver or teacher. Recent research has shown that such reading aloud to young children actually positively affects their social-emotional development (Mendelsohn et al. 2018). This is important as understanding emotional information is a skill that forms part of social-emotional development (e.g., Denham et al. 2009). Also, in face-to-face communication, people not only see another person’s emotional facial expression; they commonly also hear what the other person says and how they say it (e.g., Aviezer et al. 2017; Holler and Levinson 2019; Sendra et al. 2013). Indeed, affective prosody has shown to play an important role in the processing of facial emotional expressions (for a review, see Wieser and Brosch 2012). In both cases, the emotional context includes different sources of input that can support detecting emotion-related information.

The importance of emotion-supporting context in social interaction raises the question of whether such a context offers a setting for improved perception of emotion-related target information, such as words and faces. The present study aims to address this issue. Specifically, we examined how emotional supporting context affects the accurate detection of the emotionality of written words and facial expressions.

Previous research showed that contextual factors are -to a certain extent- automatically taken into account in emotion perception. Seemingly minimal signals such as someone’s gaze direction can -automatically- influence how emotions are perceived (Feldman Barrett et al. 2011; Mumenthaler and Sander 2015). Relatedly, voice, body posture, and a visual setting (e.g., Aviezer et al. 2017) all have been shown to affect emotion perception. More directly relevant for the present study, research supports the notion that language as well as affective voice is important contextual elements in emotion perception. Emotion words (Halberstadt and Niedenthal 2001) as well as written social labels (Mermillod et al. 2018) have been shown to affect face perception, and it is reasoned that emotion words support facial emotion perception by inducing certainty about the emotionality of such facial expressions (Gendron et al. 2012). Relatedly, not having access to an emotion word has been found to impair perception accuracy of facial expressions (Gendron et al. 2012).

Studies have also revealed the influence of voice and intonation on emotion perception. For example, a study by Rigoulot and Pell (2014) showed that vocal emotion cues influence the way in which people visually scan and process facial expressions. They presented participants with facial expressions that were accompanied by either congruent or incongruent affective prosody, which consisted of non-sensical sentences. Participants were more accurate to judge whether the face and prosody matched or not when the affective prosody matched the facial expression. Relatedly, a different study (De Gelder and Vroomen 2000b) found that affective tone of voice influences facial emotion identification. When facial expressions were presented with either congruent or incongruent affective prosody—consisting of a semantically neutral sentence—identification of the emotion in the facial expressions was biased in the direction of the presented affective prosody. The opposite also held; emotion in a face showed to influence judgment of emotion in a voice (De Gelder and Vroomen 2000b). It has been suggested that humans automatically and naturally pair affective voice and face in a successful manner (De Gelder et al. 2002), suggesting that affective voice is a natural contextual element in emotion perception.

Previous work on the influence of contextual information on emotion perception explored settings that employed combinations of affective information from multiple modalities (e.g., De Gelder et al. 2002; Rigoulot and Pell 2014), which constitutes a realistic and ecologically valid situation (e.g., De Gelder and Vroomen 2000a). It is important to note that most of these studies often compare congruent vs. incongruent situations, and demonstrate that congruent (or incongruent) settings facilitate (or undermine) accurate emotion perception (e.g., De Gelder and Vroomen 2000b; Rigoulot and Pell 2014). Whereas important and informative, these studies do not clearly reveal how emotion-supporting context improves the understanding of emotion-related information, as there is no comparison with trials in which no auditory contextual information is given, nor with trials in which auditory contextual information is of neutral prosody. In the case of visual perception of target information pertaining to written words and facial expressions in the presence of others, it is important to examine how auditory verbal cues, such as emotion-matched words and intonation of an accompanying voice enhance the accurate detection of the emotionality of the target information.

Accordingly, the present study was set out to test this systematically by assessing the contribution of each contextually supporting element of a voice (word and intonation) to the accurate detection of emotion-related target information. To increase the generalizability of our test for different modes of communication to which people are exposed in everyday life we designed two different tasks. In both tasks, emotion-supporting context was induced in the form of spoken audio. The first task compared emotion detection accuracy in written emotion-related (positive and negative) words without any contextual information to trials in which we stepwise added a pronunciation of the words and intonation that matched the valence of the written words. In the second task we focused on the influence of emotion-supporting context on emotion detection accuracy in images of emotion-related (happy and angry) facial expressions. Thus, in both tasks supporting information consisted of spoken emotional-matched words with a neutral intonation (words only), or of spoken emotional-matched words with an emotionally-matched intonation (words and intonation). Based on previous research, we expected that the emotion-supporting context facilitates the detection of emotion-related information.

Accurately detecting emotion-related information is an important ability for successful human interaction. Grounded in the embodied cognition view of social information processing (e.g., Arnold and Winkielman 2019; Niedenthal 2007; Niedenthal et al. 2005), in numerous emotion processing studies, both written words as well as facial expressions have been shown to trigger a simulation process that sometimes is reflected in facial muscle activity (e.g., Foroni and Semin 2009; Niedenthal 2007). Especially the simulation of facial expressions involves cortical processing related to motor simulation of facial expressions, the posterior cingulate cortex, and medial temporal lobe structures (Schilbach et al. 2008). For example, the smiling muscle -zygomaticus major- tends to show more activity when people perceive positive emotional information, and less activity to negative emotional information. Similarly, the frowning muscle -corrugator supercilii- tends to become activated when people perceive negative information, while showing less activity to positively valenced information (e.g., Dimberg et al. 2000, 2002). This process is also referred to as facial simulation or mimicry.

One of the main current ideas about the occurrence of facial simulation is that it serves a social function and that its occurrence depends on the social context within which stimuli are perceived (e.g., Hess and Fischer 2014). Moreover, recent views within the embodied cognition framework suggest that facial simulation can be important when emotion understanding is more complex (e.g., Arnold and Winkielman 2019; Winkielman et al. 2015, 2018 ). Some studies report the occurrence of facial mimicry as a mere response to the valence of stimuli, while not necessarily relating to emotion understanding or recognition (e.g., Blom et al. 2019). Nevertheless, other studies have in fact reported support for the notion that facial mimicry can facilitate emotion recognition and understanding (e.g., Drimalla et al. 2019; Künecke et al. 2014). In the current study we therefore also explored whether this simulation process might depend on the amount of emotion supporting contextual information one has when processing emotion words and emotional facial expressions.

## Methods

### Participants and study design

A sample of 28 students participated in this study (23 female, *M*_age_ = 21.5, *SD*_age_ = 2.41). The study had a within participants design, employing three levels of contextual auditory support: no contextual support, partial support or full support. Running a sensitivity analysis in G*Power 3.1 (α = 0.05, power = 80%, *N* = 28, non-sphericity correction *ε* = 1 and *r* = 0.5) for an ANOVA: Repeated measures indicated that we were able to detect a difference of moderate effect size between the three conditions in our experimental design, effect size *f* = 0.25. The study was conducted and written informed consent of each participant was obtained in compliance with the principles contained in the Declaration of Helsinki.

### Experimental tasks

#### Experimental task 1: emotion detection in written words

In the first experimental task, we measured accuracy of detecting emotion in written words, with different levels of contextual auditory support. Written emotional (of positive or negative valence) and neutral words were presented on screen in each trial, neutral words served as fillers. There were three levels of contextual support: (A) No contextual support (visual stimulus only), (B) Partial support by contextual information (the word presented on screen is pronounced over the headphones, with a neutral intonation), and (C) Full support (the word presented on screen was pronounced over the headphones, with emotionally matched intonation), see Table 1.

**Table 1:** The different stimulus combinations in the two tasks that apply to experimental task 1 (with visual targets being written words) and task 2 (with visual targets being facial expressions)

Contextual support level	Visual target valence	Audio information	Number of trials
(A) None	Positive	NA	12
(A) None	Negative	NA	12
(B) Partial	Positive	Positive word /Neutral intonation	12
(B) Partial	Negative	Negative word /Neutral intonation	12
(C) Full	Positive	Positive word /Positive intonation	12
(C) Full	Negative	Negative word /Negative intonation	12
			Total: 72
(A) None	Neutral (filler)	NA	24
(B/C) Partial/full	Neutral (filler)	Neutral word—Neutral intonation	48
			Total: 72

*NA* not applicable

#### Experimental task 2: emotion detection in facial expressions

The second task had a similar design as task 1, the difference being that the visual stimuli to be classified were images of facial expressions. An emotional (happy or angry) or neutral facial expression was presented on the screen in each trial, neutral expressions served as fillers. Again, three levels of contextual auditory support were used, see Table 1. For example, considering happy facial expressions, a facial expression would either (A) appear without any audio, (B) appear with the audio of a semantically positive word with neutral pronunciation, or (C) appear with the audio of a semantically positive word pronounced in a positive intonation.

### Stimuli

#### Words

Words used and tested in previous studies were selected as stimulus material.^1^ In total, 24 positive, 24 negative, and 48 neutral words were selected for the current study. Splitting these up into two separate lists enabled us to present participants with unique words in each of the two experimental tasks. Matching the 2 wordlists with the 2 experimental tasks was counterbalanced across participants.

#### Faces

Images of 6 female and 6 male actors were selected from the Dutch Radboud Faces Database (Langner et al. 2010) for task 2. Of each actor, images with a happy, negative, and neutral facial expression were used, meaning a total of 12 happy, 12 angry, and 12 unique neutral faces were used.

#### Audio

All words were read out by a professional female actor and recorded with Audacity (version 2.0.3). Positive words were recorded in a neutral and in a positive intonation, negative words were recorded in a neutral and in a negative intonation, while the neutral words were only recorded in a neutral intonation. Pretests confirmed that the intonation of these audio recordings were in line with the intended emotional intonation.

## Procedure

Upon arrival at the lab, participants were told that the study involved measurement of facial EMG to assess muscle activity during exposure to visual and audio stimuli. The EMG procedure followed the typical protocol of facial muscle activity assessment and adhered to the typical procedure and guidelines that had received approval from the ethics commission at Utrecht University. Written informed consent of each participant was obtained.

Electrodes to measure facial muscle activity were placed on the participant’s face, after which participants were seated in an individual soundproof cubicle in which the experiment took place. Participants were informed that the experiment consisted of two tasks, one in which they would be seeing words and the other in which they would be seeing faces, and that these visual stimuli would sometimes also be accompanied by audio. The two tasks were presented consecutively; the order of the tasks was counterbalanced across participants.

Participants were asked to indicate whether the stimulus on the screen was an emotional or a non-emotional one. They were instructed to be accurate and fast. Neutral stimuli served as fillers and are thus not reported in the analyses.^2^ Because each task includes an equal number of emotional and neutral targets the responses could be accurate (coded 1) or not (coded 0). Accordingly, participant’s emotion detection accuracy was assessed and served as dependent variable. Furthermore, activity of the corrugator and the zygomaticus muscles were recorded and processed for further analyses (see below at Data preparation and analysis). All visual stimuli were shown 3 times during the same task, once for each level of contextual support. Each task consisted of 144 trials, presented randomly without replacement. Written words (task 1) in partial or full contextual support were always presented with an audio pronouncing the same word, each image of a facial expression (task 2) in partial or full contextual support was presented with the audio of a word randomly chosen from the wordlist, but always semantically supporting of the valence of the facial expression.

**Fig. 1:**
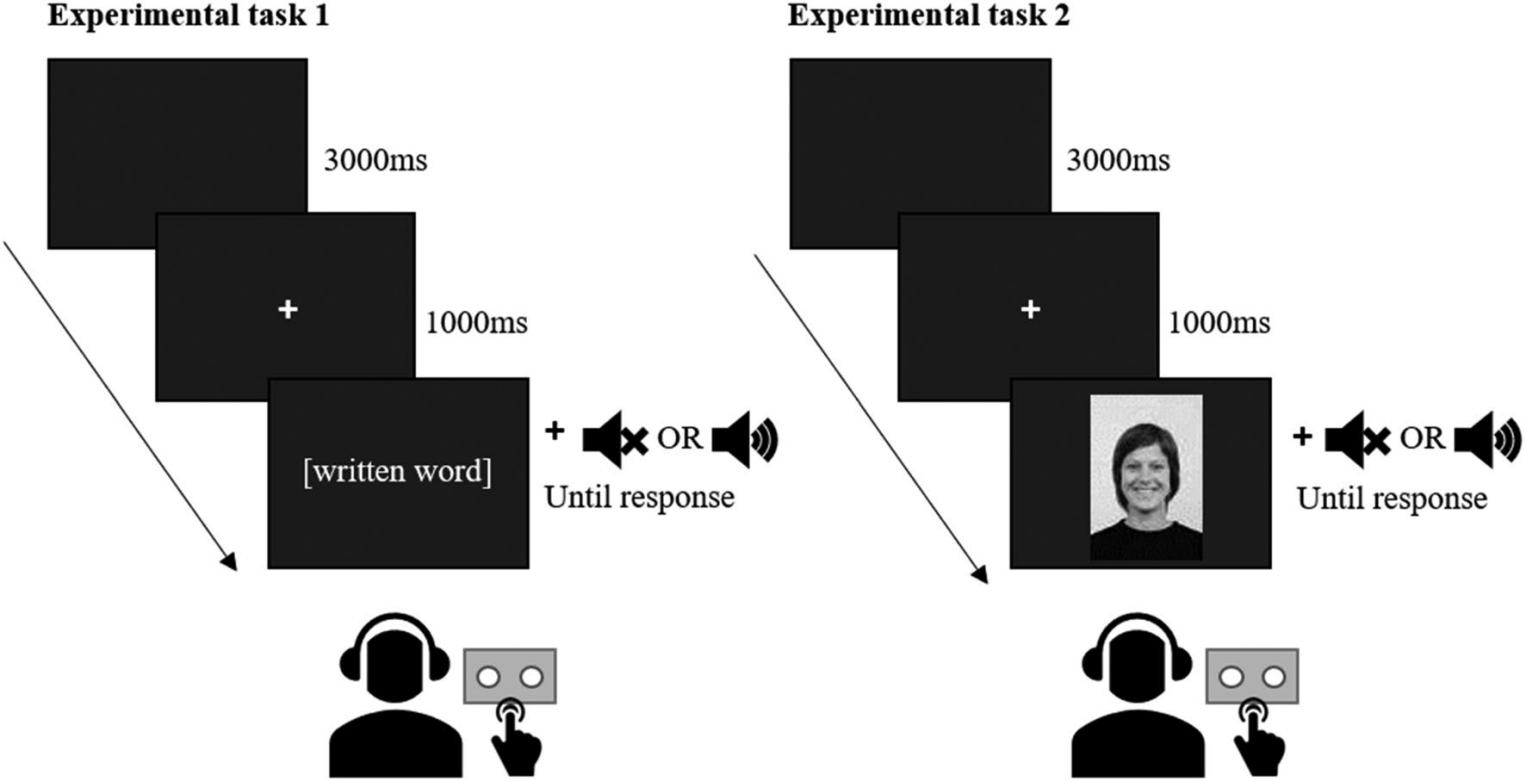
Visual overview of stimulus presentation in experimental task 1 (words) and experimental task 2 (faces)

As can be seen in Fig. 1, in each task a trial started with a blank screen (3 s), after which a fixation point appeared (1 s), followed by the visual stimulus, which remained on screen until participants classified the stimulus as being emotional or non-emotional. In the trials with partial or full contextual support, audio stimulus presentation directly followed visual stimulus presentation. Before starting each task, participants completed 4 practice trials during which participants received feedback on screen regarding their performance.

## Equipment

Participants’ responses when classifying the stimuli were recorded by use of a response box. The experiment ran on a computer with a 19-inch screen and 1280 × 1024 screen resolution. The experimental task was programmed in E-prime 2.0. Facial muscle activity was recorded and processed with MindWare Technologies EMG Application software (version 2.5).

### Data preparation and analysis

#### Behavioral measures

Emotion detection accuracy levels were calculated in percentages per stimulus type for both tasks. Positive and negative words (task 1) and happy and angry facial expressions (task 2) served as target stimuli, classifying these stimuli as emotional was considered accurate, while classifying these stimuli as non-emotional was considered inaccurate.

#### Facial EMG

Facial muscle activity at the corrugator and zygomaticus sites was measured using bipolar placements of Ag/AgCl miniature surface electrodes filled with electrode gel attached on the left side of the face. The skin was cleansed and prepared with alcohol prep pads and semi abrasive lotion. The electrodes were placed following the methods described by Fridlund and Cacioppo (1986), and all pairs were referenced to a forehead electrode placed near the midline. The raw EMG signal was measured with a BioNex Bio-Potential amplifier and stored with a sampling frequency of 1000 Hz. Raw data were filtered with a 30–300 Hz band pass filter and a 50 Hz notch filter and then rectified. Facial muscle activity recorded during the last 500 ms of each blank screen that was shown before the fixation point was used as baseline measure for that specific trial. Difference scores were calculated by using these measures as a baseline. Prior to statistical analysis, data were collapsed per type of trial and averaged over the first 1000 ms of stimulus presentation.^3^ One participant’s data were not included because of too many irrelevant facial movements due to tiredness, leading to unusable EMG measures.

### Statistical analyses

Because the two tasks used different visual targets (words or faces), we examined the behavioural data of each task separately by subjecting emotion detection accuracy levels to a repeated measures ANOVA’s with contextual support level and valence of targets as within subject variables. We first tested the main and interaction effects on emotion detection accuracy in an Omnibus ANOVA according to the experimental design. In order to gain more insight into the relationship between contextual support and emotion detection accuracy, we examine the linear and quadratic trend effect of contextual support. We furthermore examined the physiological (facial EMG with respect to the zygomaticus muscle and the corrugator muscle) data for each task. Here the effects of interest were a main effect of valence, and a possible interaction between valence and contextual support level. Though emotion detection accuracy was the main focus of the current studies, for exploratory purposes we also subjected the decision times to repeated measures ANOVA’s for each task. In addition to the frequentist statistical tests, Bayesian analyses are performed to quantify the evidence of the hypotheses under investigation (main effect of contextual support) given the data. Bayesian Factors (BF) are reported; a larger BF represents more evidence in the data set for the hypothesis under consideration. In case sphericity was violated for any of the reported results, Greenhouse–Geisser corrections were applied and adjusted degrees of freedom were reported.

### Experimental task 1: emotion detection in written words

#### Emotion detection accuracy in written words

Participants’ emotion detection accuracy levels when classifying written words was analyzed by use of a repeated measures ANOVA with contextual support level (none, partial, or full) and valence of the written word (positive vs. negative) as within participants factors.

**Fig. 2:**
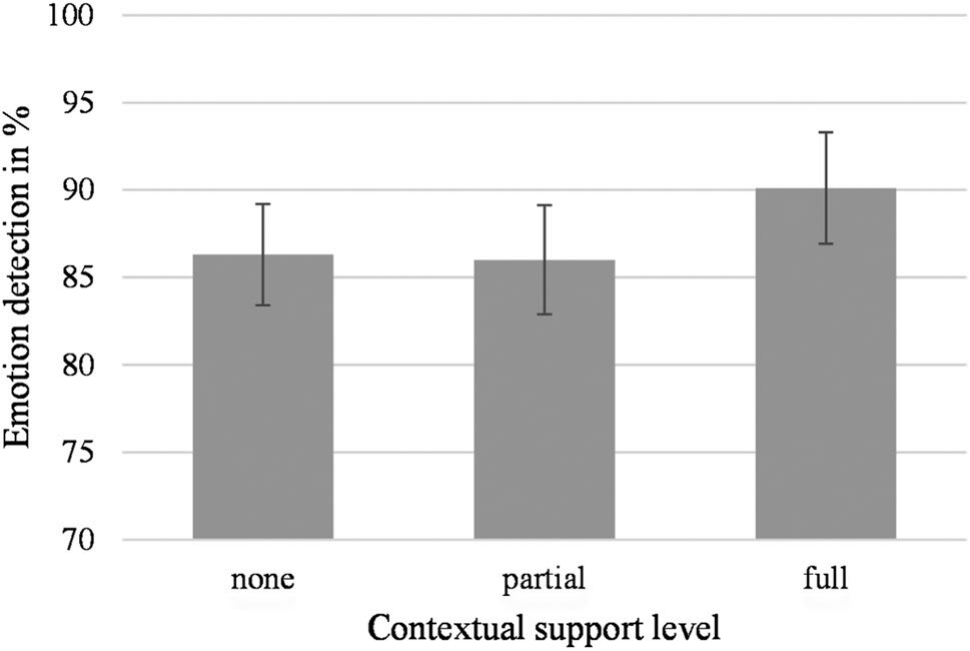
Emotion detection accuracy levels when classifying written words based on the different levels of contextual support. Error bars represent standard error

The main effect of contextual support level showed to be significant, *F*(2,52) = 4.25, *p* = 0.020, η_p_^2^ = 0.14. As can be seen in Fig. 2, highest emotion detection levels showed for fully supported written words (*M* = 90.1%, *SD* = 16.6), while partially supported (*M* = 86.0%, *SD* = 16.2) and contextually unsupported written words (*M* = 86.3%, *SD* = 15.1) had lower detection levels. A Bayesian analysis of variance showed that the data was 2.50 times more likely to reflect a main effect of contextual support level than for it not to reflect such an effect (BF_10_ = 2.50). No main effect showed for valence, *F*(1,26) = 2.32, *p* = 0.140, η_p_^2^ = 0.08. Lastly, no interaction showed between valence and contextual information *F*(2, 52) = 1.07, *p* = 0.349, η_p_^2^ = 0.04.

Furthermore, specific trend tests revealed a significant linear effect of contextual support level (*F*(1,26) = 4.78, *p* = 0.038, η_p_^2^ = 0.16), while the quadratic effect was not significant (*F*(1,26) = 3.41, *p* = 0.076, η_p_^2^ = 0.12). Finally, the analyses did not yield an interaction effect between valence and contextual support for the linear trend (*F*(1,26) = 1.18, *p* = 0.287, η_p_^2^ = 0.04) or the quadratic trend (*F*(1,26) = 1.01, *p* = 0.324, η_p_^2^ = 0.04).^4^

#### Decision times for emotion detection in written words

Participants’ decision times when classifying written words was analyzed by use of a repeated measures ANOVA with contextual support level (none, partial, or full) and valence of the written word (positive vs. negative) as within participants factors.

**Fig. 3:**
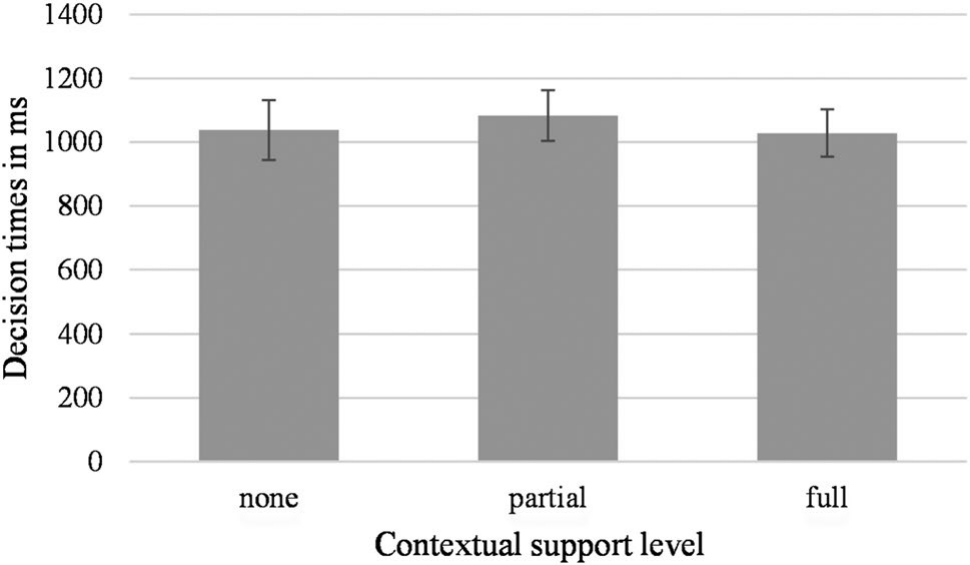
Decision times when classifying emotional words based on the different levels of contextual support. Error bars represent standard error

No effect of contextual support level showed (*F*(1.49, 35.72) = 2.03, *p* = 0.156, η_p_^2^ = 0.08), with similar decision times for contextually unsupported (*M* = 1037.9 ms, *SD* = 469.7), partially supported (*M* = 1083.1 ms, *SD* = 403.2), and fully supported (*M* = 1028.2 ms, *SD* = 371.6) written words, see Fig. 3. No main effect showed for valence (*F*(1,24) = 0.47, *p* = 0.499, η_p_^2^ = 0.02). Lastly, no interaction showed between valence and contextual information (*F*(1.31, 31.33) = 2.52, *p* = 0.115, η_p_^2^ = 0.10).

#### Zygomaticus activity to written words

Zygomaticus activity during the first 1000 ms of stimulus presentation was analyzed with a repeated measures ANOVA whereby contextual support level (none, partial, or full) and valence of the written word (positive vs. negative) were the within participants factors.

The main effect of valence on zygomaticus activity did not reach significance, *F*(1,26) = 2.92, *p* = 0.099, η_p_^2^ = 0.10. A Bayesian paired samples t-test showed that the data were 1.37 times more likely to reflect a null effect than to reflect a difference based on valence (BF_01_ = 1.37). Furthermore, the interaction between valence of the written word and level of contextual support was not significant, *F*(1.63, 42.24) = 0.54, *p* = 0.551, η_p_^2^ = 0.02.

### Corrugator activity to written words

Corrugator activity during the first 1000 ms of stimulus presentation was analyzed with a repeated measures ANOVA with contextual support level (none, partial, or full) and valence of the written word (positive vs. negative) as the within participants factors.

This analysis revealed no significant main effect of valence of the written word on corrugator activity, *F*(1,26) = 3.02, *p* = 0.094, η_p_^2^ = 0.10. A Bayesian paired samples t-test showed that the data were 1.30 times more likely to reflect a null effect than to reflect a difference based on valence (BF_01_ = 1.30). No interaction was found between valence of written word and level of contextual support, *F*(1.31, 35.10) = 0.62, *p* = 0.542, η_p_^2^ = 0.02.

### Experimental task 2: emotion detection in facial expressions

#### Emotion detection accuracy in facial expressions

Participants’ emotion detection accuracy levels when classifying the facial expressions was analyzed with a repeated measures ANOVA. Contextual support level (none, partial, or full) and valence of the facial expression (positive vs. negative) were the within participants factors.

**Fig. 4:**
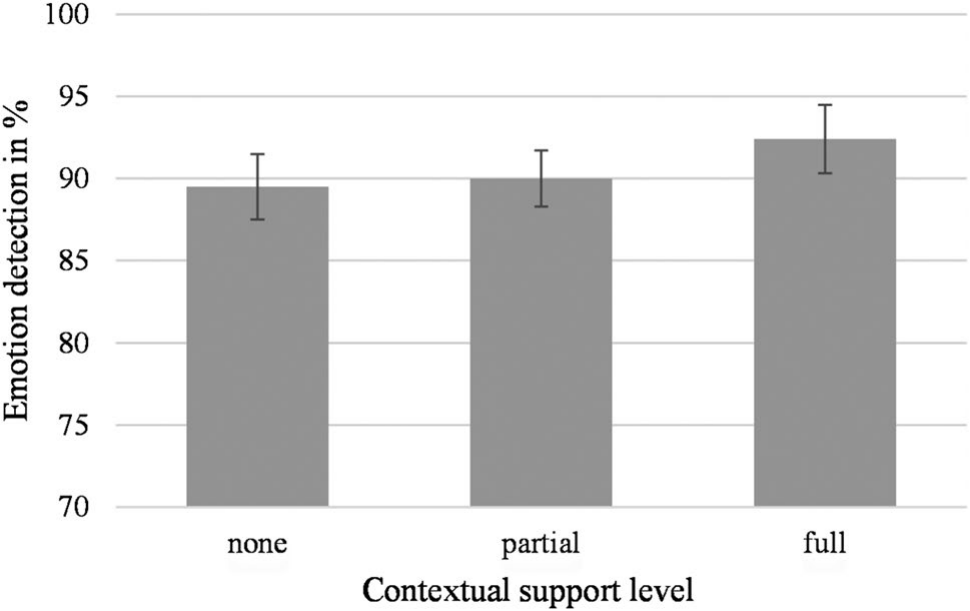
Emotion detection accuracy levels when classifying facial expressions based on the different levels of contextual support. Error bars represent standard error

This analysis revealed no main effect of contextual support level, *F*(2, 52) = 1.20, *p* = 0.309, η_p_^2^ = 0.04, see Fig. 4. A Bayesian analysis of variance showed that the data was 3.66 times more likely to reflect a null effect than for it to reflect a main effect of contextual support level (BF_01_ = 3.66). Furthermore, no main effect showed for valence, *F*(1,26) = 0.18, *p* = 0.671, η_p_^2^ = 0.01. Lastly, there was no interaction between valence and contextual support level, *F*(2,52) = 0.59, *p* = 0.559, η_p_^2^ = 0.02. Furthermore, specific trend tests revealed neither a linear effect of contextual support level (*F*(1,26) = 1.18, *p* = 0.287, η_p_^2^ = 0.04), nor a quadratic effect (*F*(1, 26) = 0.16, *p* = 0.696, η_p_^2^ = 0.01). Finally, the analyses did not yield an interaction effect between valence and contextual support for the linear trend (*F*(1,26) = 1.15, *p* = 0.294, η_p_^2^ = 0.04) or the quadratic trend (*F*(1,26) = 0.24, *p* = 0.878, η_p_^2^ = 0.00).^5^

#### Decision times for emotion detection in facial expressions

Participants’ decision times when classifying facial expressions was analyzed by use of a repeated measures ANOVA with contextual support level (none, partial, or full) and valence of the facial expression (positive vs. negative) as within participants factors.

**Fig. 5:**
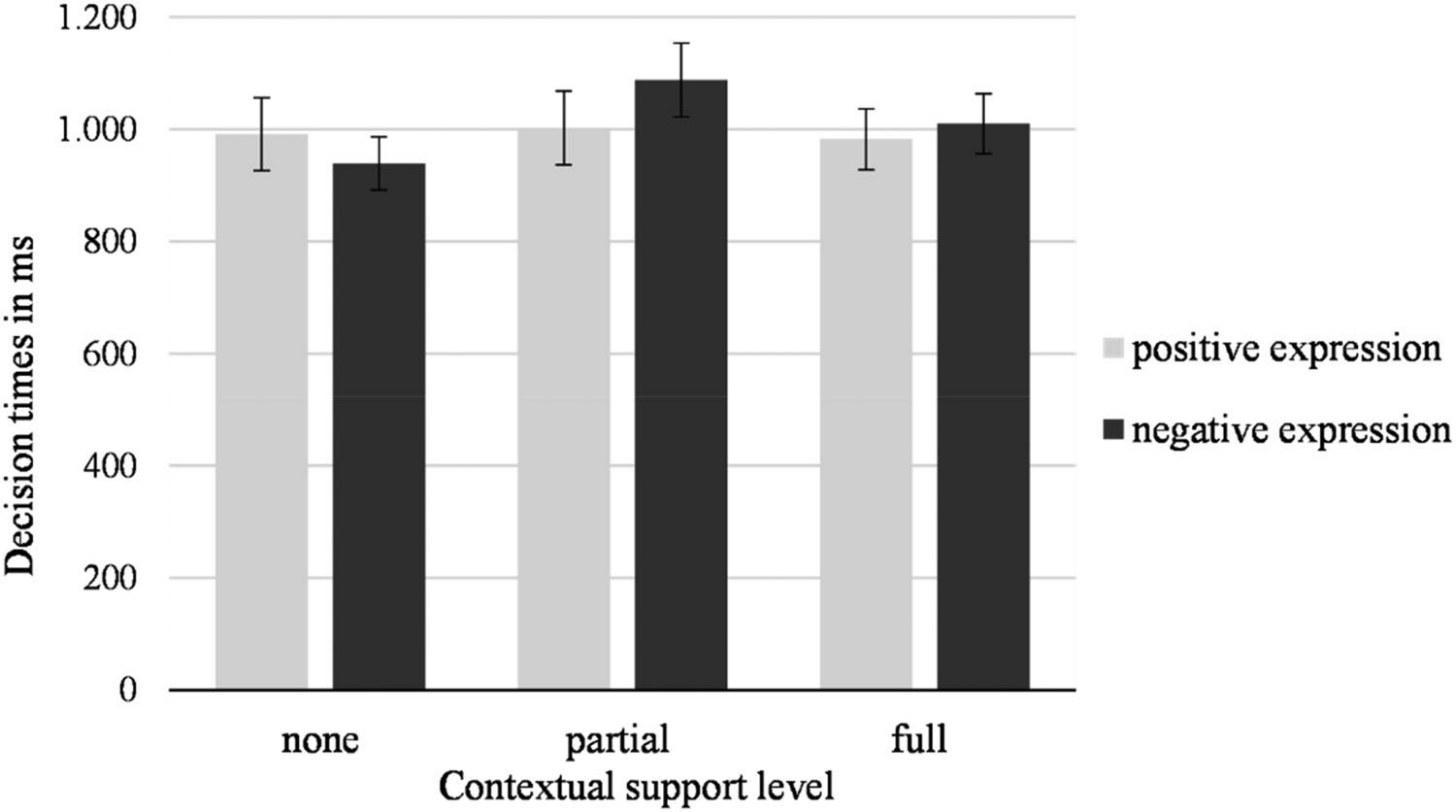
Decision times for classifying facial expressions based on the different levels of contextual support. Error bars represent standard error

The main effect of contextual support level was significant (*F*(2,52) = 4.22, *p* = 0.020, η_p_^2^ = 0.14). Decision times for faces that were partially supported contextually were longest (*M* = 1045.0 ms *SD* = 284.0), while decision times for contextually unsupported (*M* = 965.3 ms, *SD* = 316.7), and fully supported (*M* = 996.3 ms, *SD* = 268.6) facial expressions were shorter. No main effect showed for valence (*F*(1,26) = 0.56, *p* = 0.462, η_p_^2^ = 0.02). Lastly, the interaction between valence and contextual information was also significant (*F*(2,52) = 4.73, *p* = 0.013, η_p_^2^ = 0.15) = 0.10. As can be seen in Fig. 5, the difference seems to occur in decision times for negative facial expressions, but not for positive facial expressions, with longer decision times for context supported negative facial expressions.

#### Zygomaticus activity to facial expressions

Zygomaticus activity during the first 1000 ms of stimulus presentation was analyzed with a repeated measures ANOVA. Contextual support level (none, partial, or full) and valence of the facial expression (positive vs. negative) were the within participants factors.

**Fig. 6:**
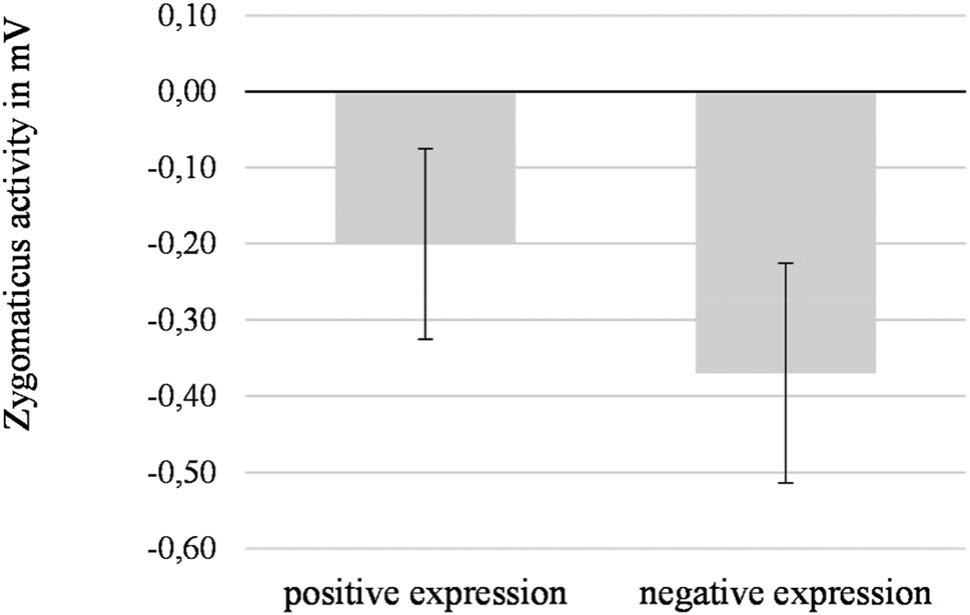
Zygomaticus activity to positive and negative facial expressions. Error bars represent standard error

This analysis revealed a main effect of valence of the facial expression, *F*(1,26) = 4.56, *p* = 0.042, η_p_^2^ = 0.15, see Fig. 6. In line with expectations, zygomaticus activity was stronger when participants saw positive (*M* = − 0.20 mV, *SD* = 0.65) than when they saw negative facial expressions (*M* = − 0.37 mV, *SD* = 0.75). A Bayesian paired samples t-test showed that the data were 1.43 times more likely to reflect such difference than to reflect a null effect (BF_10_ = 1.43). No interaction was found between valence of the facial expression and level of contextual support *F*(1.19, 30.94) = 2.95, *p* = 0.090, η_p_^2^ = 0.10.

#### Corrugator activity to facial expressions

Corrugator activity during the first 1000 ms of stimulus presentation was analyzed with a repeated measures ANOVA. Contextual support level (none, partial, or full) and valence of the facial expression (positive vs. negative) were the within participants factors.

No main effect of valence of the facial expression was found for the corrugator; *F*(1,26) = 2.83, *p* = 0.105, η_p_^2^ = 0.10. A Bayesian paired samples t-test showed that the data were 1.42 times more likely to reflect a null effect than to reflect a difference based on valence (BF_01_ = 1.42). No interaction between valence of the facial expression and level of contextual support was found*, F*(1.17, 30.45) = 0.04, *p* = 0.877, η_p_^2^ = 0.00.

## Discussion

The current study included two tasks to assess the role of contextually supportive elements of a voice—word and intonation- in accurately detecting emotion-related information in written emotion-related words and in facial expressions, relating to two common forms in which people encounter affective information in everyday life. Furthermore, we explored whether adding contextually supportive elements of a voice would relate to differences in facial simulation. Our results showed increased emotion detection accuracy levels when adding contextually supporting voice elements for written words, but not for facial expressions. Furthermore, we found some evidence for facial simulation effects in response to observing facial expressions, an effect that was not qualified by contextually supporting voice elements.

Our findings support previous work on the relevance of contextual information for emotion processing (e.g., Feldman Barrett et al. 2011; Mumenthaler and Sander 2015). Specifically, the observation that different elements of supporting contextual voice elements can affect the detection of emotion-related information concurs well with the informative element of the human voice (e.g., Aviezer et al. 2017); semantical information and intonation elements improved emotion detection in written words, underlining the advantage of multimodal information in emotion processing (e.g., Paulmann and Pell 2011). This is central to an embodied cognition perspective to social information processing according to which sensorimotor processes in dedicated cortical areas are involved in simulating the emotional meaning of such information (e.g., Niedenthal 2007; Niedenthal et al. 2005).

The fact that contextual information did improve emotion detection for written words but not for facial expressions could be due to several reasons. First of all, as emotion detection accuracy was rather high for facial expressions compared to the written words, it is possible that a ceiling effect occurred for emotion detection in facial expressions. Second, the word that was presented verbally as part of the contextual support manipulation mapped exactly on the written target word task while this was not the case for the facial expression task, where the verbally presented words only affectively mapped on the target faces. These differences in methodology lead to differences in information mapping. Consequently, it might have been the case that the facial expression task (compared to the word task) was somewhat more challenging and confusing to participants, especially when they had to process an emotionally matched word pronounced with a specific intonation while seeing the facial expression. This idea is supported by the differences in decision times between contextual support levels in the facial expression task, while these differences did not show up in the word task.

Importantly, the absence of improved emotion detection accuracy for facial expressions does not mean that emotion detection of faces cannot benefit from contextual information. Such effect might be more likely to occur when the contextual information mapped more directly on the facial expressions, such as words (e.g., happy or angry) that denote the facial expressions. Hence, future studies could utilize an improved design that would as such render it possible to clearly examine the possible added value of contextual information for emotion detection in facial expressions. This is however speculative and requires further research. Here, we would like to stress that the current study only examined a small subset of emotions, and it would be important to examine the role of contextual information in emotion detection and processing of other emotions.

Interestingly, while facial simulation did reflect the valence of the facial stimuli, it did not interact with the differences in level of contextual support. The fact that contextual support did not lead to differences in facial mimicry suggests that, at least in the current study design, contextual information is not automatically reflected in facial mimicry. Whereas this might appear to contradict the view that cognition and emotional meaning is grounded in sensorimotor processes, there is research suggesting that facial simulation does not always relate to or occur in emotion processing tasks (e.g., Arnold and Winkielman 2019). Various routes to emotion processing have been shown to play a role in detecting and grasping emotional concepts (e.g., Arnold and Winkielman 2019; de la Rosa et al. 2018; Stel 2016). In the present study such routes pertain to visual information, auditory information, semantic information as well as previous knowledge on emotion concepts. Our findings, then, suggests that facial simulation in general, and mimicry in particular, is likely to be more implicated in the valence of stimuli, but not necessarily involved when linking contextual information to the valence of stimuli in the task at hand.

The present study indicates that contextually supporting voice elements can facilitate the detection of emotion-related information, as is revealed by higher accuracy of classifying written emotional words as actually being emotional. However, it is important to note that, in the present task, contextual supporting information might also have increased the intensity of the emotional target information and therefore participants were better in detecting the emotional meaning (e.g., Montagne et al. 2007). In that case, accuracy does not represent detection quality, but strength of emotional experiences. Whereas we cannot rule out this alternative account empirically, our general findings suggest that strength of emotional experiences is not the sole cause of our effects. First, we only observed context supporting advantage for emotion detection accuracy in the case of written words, and not for facial expressions. Furthermore, our results showed that facial mimicry only depended on the valence of stimuli, and thus was not further enhanced by adding contextual supporting emotional information. Together, these findings are not easy to explain with a general strengthening account of emotional experiences of the target stimuli. Whereas detection of emotional stimuli and intensity of emotional experiences have been linked to neural networks that play a different role in social cognition and behavior (e.g., Rapport et al. 2002), it is important for future research to more clearly examine how contextual supporting information impinges on the perception of emotions when processing words during reading and facial expressions of others in interactions.

Ruling out this issue is particularly important, because it bears on real-life situations where one sees someone’s facial expression while also hearing her speak, or a caregiver or teacher read aloud to a child with supporting intonation hereby aiding in detecting emotion. In such cases, being able to correctly detect and respond to emotions can make the difference between a smooth and stiff social interaction (e.g., van Kleef 2016). For example, the role of supporting voice elements could relate more to signaling the fact that one should pay attention (e.g., Brosch et al. 2008; Sander et al. 2005; Vuilleumier 2005; Wegrzyn et al. 2017), which could also positively influence interaction and communication by taking notice when the other person is portraying positive or negative emotions with different intensity. Thus, while we reason that added voice elements could be argued to positively influence daily life interactions, we need more research to further explore the mechanism that allow people to include auditory context in processing emotional information conveyed by interaction partners.

## Data Availability

The data set is available and stored and can be requested from the corresponding author.

## Appendix

See Table

**Table 2:** Words (translated from Dutch) selected for this study

	Positive words	Negative words	Neutral words	
Bradley and Lang (1999)	Friendly Nice Thoughtful Adorable Cute Hopeful Pleasure Reward Cuddle	Disgusted Upset Morbid Cruel Selfish Insane Terrible Rotten Aggressive	Contents Avenue Clock Doctor Context News Stove Month Obey Gloom Radiator Passage Tool Glass Appliance	Icebox Ankle Time Hat Hammer Trunk Stool Material Elevator Scissors Knot Office Rattle Truck Odd
Foroni and Semin (2009)	To smile To laugh To grin Comical Funny Entertaining	To frown To cry To squeal Irritating Frustrating Annoying		
Hermans and De Houwer (1994)	Healthy Happy Fortunate Optimistic Cheerful Pleasant Love Peace Hug	Hateful False War Cancer Torture Accident Pain Sadness Death	Table Page Branches Circle Clay Paper Box Plate Bag	Wallpaper Sidewalk Stripe Square Sewing machine Bow Microscope Yeast Line

2.

## Footnotes

1 Six positive and six negative Dutch words previously used in a study by Foroni and Semin (2009), and 9 positive, 9 negative, and 18 neutral Dutch words from a study reporting their emotional meaning and perceived valence (Hermanns and De Houwer 1994) were selected. Lastly, selected words from a study reporting affective norms of English words (Bradley and Lang 1999) were translated into Dutch and pilot tested for the current study on their valence and emotionality. Of these words 9 positive, 9 negative, and 30 neutral rated words were selected. See Appendix I for the complete list of words selected for this study.

2 For task 1 (visual stimuli written words), average accuracy levels for written words supported by contextual information -audio with neutral intonation- was 96.6% (*SD* = 3.3), and for words not supported by any contextual information accuracy was 95.3% (*SD* = 2.6). For task 2 (visual stimuli facial expressions), average accuracy levels for faces supported by contextual information -audio with neutral intonation- was 91.2% (*SD* = 11.4), and for faces not supported by any contextual information accuracy was 94.6% (*SD* = 11.3).

3 The first second of seeing the stimulus is chosen as it can be assumed that facial muscle activity during this time window is considered to be spontaneous, while after the first second more deliberate processes can occur (Häfner and IJzerman 2011).

4 We also inspected the possible effect of contextual information on the detection of neutral words. Neutral words were always presented either without any contextual information, hence it was not possible to test the full design for neutral stimuli. A small difference in detection of non-emotionality showed for neutral words, *t*(23) = 2.80, *p* = .010. Neutral words without contextual information were detected somewhat less accurate (*M* = 95.3%, *SD* = 2.55) than neutral words with contextual information (*M* = 96.6%, *SD* = 3.30).

5 No difference in detection of non-emotionality showed for neutral facial expressions, *t*(26) = 0.54, *p* = .597. Neutral faces without (*M* = 94.6%, *SD* = 11.25) and with contextual information (*M* = 94.1%, *SD* = 11.38) were detected equally accurate.
